# Family members’ expressions of dignity in palliative care: a qualitative study

**DOI:** 10.1111/scs.12913

**Published:** 2020-10-06

**Authors:** Anna Sandgren, Lena Axelsson, Tove Bylund‐Grenklo, Eva Benzein

**Affiliations:** ^1^ Department of Health and Caring Sciences Linnaeus University Växjö Sweden; ^2^ Center for Collaborative Palliative Care Department of Health and Caring Sciences Linnaeus University Växjö Sweden; ^3^ Department of Nursing Science Sophiahemmet University Stockholm Sweden; ^4^ Department of Caring Science University of Gävle & Department of Oncology Pathology Karolinska Institutet Stockholm Sweden

**Keywords:** dignity, family members, interviews, palliative care

## Abstract

Living and dying with dignity are fundamental values in palliative care, not only for the patient but also for family members. Although dignity has been studied from the different perspectives of patients in need of palliative care and their family members, family members’ thoughts and feelings of dignity have not been given sufficient attention. Therefore, the aim was to describe family members’ expressions of dignity in palliative care. The study had a qualitative design; semi‐structured individual interviews were conducted with 15 family members of patients in palliative care in a county with a specialist palliative advisory team. Data were analysed using inductive content analysis. The results showed that family members’ expressions of dignity are multifaceted and complex. For family members in palliative care, dignity means living as a respected human being in relation to oneself and others. Dignity also includes being able to maintain one’s identity, feeling connected to significant others, and being comfortable with the new situation. Two contextual aspects affect family members’ dignity: the two‐headed paradox and reciprocal impact. The *two‐headed paradox* means that family members want to stay close to and care for the ill person, at the same time want to escape the situation, but when they escape, they want to be close again. *Reciprocal impact* means that family members’ feelings and experiences of the situation are closely intertwined with those of the ill person. These results may increase healthcare professionals’ understanding and be used in dignified care practices that do not threaten, but instead aim to preserve family members’ sense of dignity.

## Background

Living and dying with dignity are fundamental values in palliative care for both patients and their family members (1). The situation for family members of a seriously ill person is often demanding in both the practical and emotional dimensions of everyday life (2, 3). This has an impact on how family members uphold their dignity. Although the concept of dignity is complex, Nordenfelt (4) offers four notions of dignity to provide further understanding. He argues that all humans have dignity that cannot be lost as long as we live, that is a universal human dignity. Nordenfelt’s other three notions of dignity can fluctuate and be affected by oneself and others. He claims dignity of merit exists in degrees and is related to one’s status in society; dignity as moral stature is linked to one’s moral values and self‐respect. The fourth type of dignity is the dignity of identity, which is linked to a person’s identity as a human and to one’s integrity of body and mind. Our dignity of identity can be violated or taken from us by things such as the acts of other persons or illness.

In a review synthesising the meaning of dying with dignity, it was found to be a process where the significance of intrinsic (related to self) and extrinsic (related to others) aspects of dignity change over the course of advancing illness (5). Another review explored the transition experience of family members of persons with advanced disease and showed a life transition involving a redefinition of life in order to maintain hope and personhood as life is altered. This process is strengthened by supportive factors such as receiving information and having positive attitudes towards their caregiving (6). How this life transition may influence family members’ perspectives of dignity in palliative care has been scarcely reported. It has, however, been found that family members may feel responsible for ensuring that the patient has a dignified death (7) and that they find dignity becomes increasingly important towards the end of life (8). A growing need for care may also be a major threat to dignity and, as illness progresses, the quality of care given is increasingly significant for patients’ dignity (5). In Guo and Jacelon’s review of 52 studies about dignity (5), family members’ views were included in only four studies, and none focused solely on family members or their own dignity. However, the integrated findings of family members’ perspectives showed that they regard symptom relief, autonomy, independence, respect, being human, self‐image and role preservation as important aspects of dignity for dying patients. A study of the lived experience of intersubjective dignity from a dyadic perspective from dyads living with serious illness in a palliative context showed that being available and upholding continuity were central themes (9). Today, it is increasingly common that family members have an extensive caring responsibility (10) and may act as both caregivers and care recipients (11). Their increasing involvement with and responsibility for the patients signifies that they will have an increasing role in the palliative care (11). Despite these findings, research on family members’ thoughts and feelings of dignity in this context are still warranted. Family members’ experiences of their own dignity are important, since providing dignified care encompasses the family (5). Therefore, this study aims to describe family members’ expressions of dignity in palliative care.

## Methods

### Setting and participants

This study had a qualitative descriptive design with an inductive approach to capture the expressions of the family members. It was conducted in a medium‐sized county in southern Sweden with a population of almost 187 000 people. The county did not have any 24‐hour specialist palliative care services or palliative care units. A specialist palliative advisory team served the population during daytime hours Monday to Friday and functioned as a link between the hospitals and community‐based home‐care nursing, supporting healthcare professionals in palliative care issues.

Inclusion criteria for the participants were that they must be family members of patients in need of palliative care, aged 18 years or older, and able to understand and speak Swedish. The sample was guided by a purposeful sampling (12) in order to reach a variation in ages and relationships to the patients. The family members were mostly recruited through the patients, but some were recruited directly by the nurses when the patients were not able to collaborate. Nurses gave oral and written information about the aim of the study to the patients and/or their family members and asked if the family members consented to being contacted by phone by a researcher. The researcher gave further information about the study. All the contacted family members agreed to participate, and the date, time and place for the interviews were set according to their wishes. In total, 15 family members participated in the study, twelve women and three men, aged 26–93 years. They were all close family members of an ill person, such as parents, spouses or children. Most of the patients suffered from cancer, while others had been diagnosed with amyotrophic lateral sclerosis or multiple sclerosis. Most patients were connected to the specialised palliative advisory team.

### Data collection

Data was collected between May 2015 and March 2016, through interviews by three of the authors (AS, TBG, EB), all experienced interviewers. The majority of the interviews were performed in participants’ homes and some were performed in the hospital. A semi‐structured interview guide with open‐ended questions was used to encourage participants to talk about dignity in relation to themselves as family members. Examples of questions were ‘Can you tell me what dignity means to you? How can you live a dignified life?’ and ‘What characterizes dignified care?’ Follow‐up and probing questions were used to gain rich data, such as Can you explain what you mean by that? Can you give an example? The interviews lasted between 26 and 115 minutes (approximately 25 hours in total) and were audio‐taped and transcribed verbatim.

### Data analysis

The interviews were analysed using inductive content analysis (13). Each author read through each interview several times to become familiar with and acquire a preliminary understanding of its content. Meaning units corresponding to the study aim were then identified by two of the authors (AS, EB). The meaning units consisted of one or several sentences or parts of text and were extracted and written into an analysis matrix, where they were condensed, abstracted and given codes. The coding was performed after thorough discussion of the analysis to ensure no meanings were lost. The codes were compared to identify similarities and differences and, based on this comparison, abstracted into sub‐categories. The sub‐categories were discussed and revised several times, then abstracted into categories. Finally, the authors inductively formulated the underlying meaning of the categories into an overall theme. Throughout the whole analysis process, all authors met to discuss and compare the analysis with the collected data.

### Ethical considerations

The study was approved by the Research Ethics Committee in Linkoping, Sweden (Nr 2014/304‐31). In accordance with the ethical guidelines in the Declaration of Helsinki (14), participants received oral and written information about the study aim, the voluntary nature of participation, the right to refrain at any point in time without having to specify why, and that the study was confidential. All the participants signed an informed written consent before the interview.

## Results

Family members talked about their dignity in an engaged way. Although the focus in the interviews was on family members’ own experiences, their stories often included expressions of how the ill person’s experience of dignity had a large influence in relation to their own dignity. As both perspectives often were intertwined, the results also enclose expressions of intersubjective dignity.

### Living as a respected human being in relation to oneself and others

Family members’ expressions of dignity inductively interpreted as the overall theme ‘Living as a respected human being in relation to oneself and others’. Two contextual aspects emerged as important for understanding the nuances of their expressions (Table 1).

**Table 1:** Overview of the results

Living as a respected human being in relation to oneself and others		
Being able to maintain one’s identity	Feeling connected to significant others	Being comfortable in the situation
Being invited to tell one’s story	Doing something good	Living as fully as possible
Being appreciated for one’s knowledge	Being able to share similar experiences	Feeling a sense of at‐homeness
Maintaining one’s own persona	Receiving support and encouragement from healthcare professionals	Receiving desired support
**Contextual aspects influencing family members’ dignity** Two‐headed paradox Reciprocal impact		

### Contextual aspects influencing family members’ dignity

These aspects should be seen as family members’ living circumstances, which have a profound impact on how family members experience and express their dignity. The first contextual aspect was that being a close family member in palliative care is a *two‐headed paradox*. On the one hand, they wanted to be close to and care for their ill family member; on the other hand, they also wanted to escape. When they did escape, they wanted to be close again. Further, although they truly wanted to care for their family member, they also felt that they were obliged to do it and had no choice. This paradox is a constant struggle that family members must cope with. Another second aspect was the *reciprocal impact* between the family member and the ill person. This was illustrated by expressions such as ‘If I see that he (ill husband) is feeling well, then I feel well’ or ‘When she (ill wife) is satisfied with the care, so am I’. This reciprocity indicates that the family members’ feelings and experiences are closely intertwined with those of the ill person. This symbiosis points to the close interpersonal relationship between the family member and the ill person. As a consequence, family members’ expressions of dignity are closely tied to and effected by how the ill person experiences the situation and his/her dignity.

### Being able to maintain one’s identity

*Being invited to tell one’s story* is linked to maintaining one’s identity, a prerequisite for feeling acknowledged. Narrative is a means of putting thoughts and feelings into words to make sense of a situation. The family members emphasised the importance of healthcare professionals actively listening to their story. These stories should form a foundation both for the support they receive and for the care of the ill person. They further argued that when professionals truly listened, family members became comfortable and their sense of dignity was supported. When professionals did not ask about the family members’ or the ill person’s stories, they felt betrayed and abandoned, as if both their own and the ill person’s sense of dignity had been violated.

*Being appreciated for one’s knowledge* occurs when family members are asked to share their knowledge of the ill person’s illness. They wanted to be seen as intellectual beings, not just as family members. They argued that they had extensive competence and wanted to share their knowledge about the ill person; since they knew the ill person better than the professionals. However, their knowledge was often neglected and they felt questioned and were not invited to participate in meaningful conversations. When family members felt they were being ignored and diminished by the professionals, it felt as if they were being treated without respect. They also often felt disrespected when professionals acted as if they ‘knew without knowing’. One woman said:

I think it is, above all, about dignity and personal treatment, about how the family members should be treated and that they should be listened to, always, they always have something to give, this shouldn’t be dismissed, I’ve said this, I know that I’ve said this to numerous nurses and doctors, make use of me, I’m sitting on so much knowledge about this person, ask me, use me.

*Maintaining one’s own persona* is crucial for a sense of dignity. However, it is only possible when one’s identity, personalities and roles are acknowledged by others. Having a positive outlook on life facilitated feeling a sense dignity, as opposed to having a negative outlook. Further, family members wanted to maintain their roles as, for example daughters and wives, and not become formal caregivers. However, they realised that some role changes were inevitable and something they had to adjust to, which meant keeping up a facade for themselves, the ill family member, and those around them. When they felt forced to take on unwanted tasks and roles, their sense of dignity was jeopardised. One wife said:

I shower him and I dress him, he can’t bend down so I have to put his socks on him and as soon as he moves it is so painful for him, so I have to help him with everything, wash him, so then I take on some kind of role and this is difficult sometimes… I’m actually his wife, that’s what I am. I think a lot about that, then I get upset, because being a wife, that’s gone.

The family members emphasised situations when both their own and the ill person’s dignity was violated; for example, when professionals addressed them in a rude or disrespectful way and they were treated ‘like a child’. This was the ultimate way of ignoring the family members’ own persona and violating their sense of dignity.

### Feeling connected to significant others

*Doing something good* permeates the family members’ relationship to the ill person. This close relationship is foundational for their sense of dignity, but also for being worthy of their mission to care for the ill person. They had to adapt to being the ill person’s spokesman. Often, spouses made agreements to take care of each other in their home and fulfilling that promise strengthened their sense of dignity as individuals and as couples. Giving the ill person ‘permission’ to die was considered a respectful way of showing their love and concern. Passing on memories, such as significant activities, narratives and souvenirs, to the coming generation also supported their sense of dignity. One wife talked about her grandchild:

We have had a dignified life and have been able to pass that on, as one says. Then our grandchild, when I was home with him, asked: will granddad ever get better? No, I’m afraid not, I said. And he remembered when his granddad jumped on the trampoline, he has memories of his granddad.

However, even if this close relationship was a source of energy, it could also be energy‐draining. Family members sometimes thought the bonds to the ill person were too strong, making them feel alone and that their own living space had become limited. These dissonant feelings made family members feel uncomfortable. Being connected with close and extended family was, therefore, a prerequisite for family members’ sense of dignity.

*Being able to share similar experiences* with other family members and the extended family was very important. They could make shared decisions and support each other through open communication. It was comforting to share experiences with extended family who had experienced similar life situations with a dying family member. Sharing experiences also influenced their thoughts about what constituted a sense of dignity. Close family became more important as end‐of‐life approached.

*Receiving support and encouragement from healthcare professionals* is of utmost importance for family members’ sense of dignity, as well as for the ill person. It is crucial that professionals show concern and acknowledge family members as caring partners, making decisions about care in collaboration with the family members. They were often very sensitive to encounters with professionals, since their feelings of hope were nurtured by good relationships. One mother of a dying daughter narrated her experiences of a nurse who acknowledged her needs of closeness and comfort in the middle of a medical technical environment, supporting her sense of dignity in the situation:

Every time I was sad or tired or something, she (the nurse) would move my daughter a little to one side to make some space between all the leads and wires so that I could crawl in and lie beside her, and there were not many people who would have done that, but she did; she was a wonder of human kindness.

Hope was closely linked to the future and, as long as family members could feel hope, the future was considered to be meaningful. The encounters with the palliative advisory team were often emphasised as supportive, and the family members had trust and confidence in their competence. Their sense of dignity was violated when the professionals ignored or humiliated them, such as when they did not show up at the agreed time or talked over their head or that of the ill person. They argued that things like inadequate staffing and lack of continuity and competence profoundly impacted the quality of the relationship and their sense of dignity. Sometimes, family members felt there was an ‘us’ and ‘them’ situation, saying that the professionals sometimes had a collegial solidarity that hampered the relationship with the family and their sense of dignity.

### Being comfortable in the situation

*Living as fully as possible* together in the present situation facilitated a sense of dignity. Family members struggled to maintain independence. This struggle involved both their own independence and the independence of the ill person, which had to be balanced despite their interdependence. Some said that if you cannot do the things you want to do, it is not a life with dignity. Family members believed that the ill person’s loss of control over bodily functions was not automatically a threat to their own and/or the ill person’s senses of dignity. However, when the care needs of the ill person were not acknowledged and treated, this could influence both family members’ and the ill persons’ sense of dignity, because they were interconnected.

*Feeling a sense of at‐homeness* is facilitated if choosing the place of care is a shared decision between the ill person, themselves, and healthcare professionals. Being in a familiar, secure and calm milieu increased the feeling of at‐homeness for all involved. Home was often considered to be a good place for dying and death. Another prerequisite for a sense of dignity in relation to place of care was that family members received the support they needed. One woman said:

We organized everything at home with his pillows, and he could run up and down when he wanted, and light the fire, because he was often freezing cold; yes, the fire was alight day and night. No, it was no problem, home is where he felt safe and secure.

There were, however, times when family members’ sense of dignity was violated. They argued it was sometimes exhausting to have a large number of professionals in the house every day. They had to relate to a variety of people, and they often had to change their daily routines to fit around the professionals’ schedules. Further, it was sometimes expressed as isolating, since they always had to be available for both the ill person and for the professionals. Their home was both literally and existentially transferred from being a home to being solely a place of care, something which hampered their feeling of being ‘at home’ and, thus, their sense of dignity.

*Receiving desired support*, such as having someone to call when the situation became too challenging increased their sense of dignity. When the professionals did ‘small things’, such as giving them a call just to check in or passing by even though they had no scheduled appointment, family members were extremely grateful. Knowing where and to whom they could turn to get support, created a sense of security and fostered their sense of dignity. One woman said:

I received a lot of support from the advisory team, as soon as I rang it was no problem, I was given help straight away, I could ring several times a day, which was never questioned, no, it was really of enormous support to us.

Having the possibility for respite was necessary to replenish energy levels and endure the situation, which, in turn, facilitated their sense of dignity. The respite helped family members to escape from the ‘caring bubble’, in which they often felt trapped. However, some family members did not dare to leave their home, as they believed that the care for the ill person would then be neglected. When hospital care was needed, they said that the possibility to enter the hospital ward directly, without first spending time at the emergency department, facilitated a sense of dignity for both the ill person and the family member. When the ill family member was admitted to hospital or another place of care for a couple of days, family members could feel free, at least for a short while.

## Discussion

This study showed that family members’ expressions of dignity were multifaceted and complex. The two‐headed paradox meant that family members can be seen with a constant struggle between being happy to care for the ill person and simultaneously feeling that they are trapped. This is in line with earlier research showing that family members express comfort when they are able to help and guilt when they feel that their capability is inadequate (15). Breen et al (16) highlighted the complex experience of feeling gratitude to be able to care for an ill family member and even consider caring as a gift to the ill family member, while also feeling that the caring situation consumed every part of their life and deprived them of their normal daily routines. Reciprocal impact is like being closely intertwined in a symbiosis‐like relationship, as shown in this study. The importance of reciprocal relationships has been described in earlier research. Wright and Leahey (17) suggest that being in a reciprocal relationship can be seen as a normal way of relating to each other. In such interactions, intersubjective or social dignity is created. This kind of dignity is explored in a study of dyads living with serious illness (9). They found that how to respond and being responded to, as well as maintaining emotional bonds and valued activities in daily living within the dyad, are important features to maintain intersubjective dignity. De Brito et al (18) also suggest positive outcomes from reciprocity in relationships, such as increased psychological well‐being in older persons, while Kihlgren, Blomberg, and James (19) suggest that a reciprocal relationship is an opportunity and a solution for a meaningful daily life, as based on studies involving older persons in need of home care. However, the family members in our study talked about the potential negative aspects of reciprocity, that is when the relationship becomes too close, it makes them feel that they are not an independent person. It has been shown earlier that the health of family members is affected negatively when they are living with a person with cancer (20) and that the patterns of familial interaction are complex and may change in palliative care (21).

Family members expressed living as a respected human being is vital in order to uphold one’s dignity. This goes in line with how patients strive to maintain their dignity by being treated as a person of value (22). This can be understood in accordance to Nordenfelt’s (4), assertion that universal human dignity is inherent for all human beings and cannot be lost, although it needs to be respected. This was obvious for the family members in our study, as they wanted to be met as a respected human, not just as one family member among all the rest. Therefore, it is vital that the care and care environment of severely ill persons should enable feelings of and maintenance of the individual’s dignity (23).

Our results also show that maintaining one’s identity is important for maintaining a sense of dignity. Identity was connected to being able to tell one’s story to someone who truly listened. This result can be compared to a study by Barclay (24), who suggests that when professionals fail to respect dignity, there is a risk of harming people. This can be further understood by Nordenfelt (4), when he points out that dignity of identity can be violated by others. Thus, when family members feel they are not invited by the healthcare personnel to tell their story, their dignity of identity is violated and often, also, their self‐image. Further, when family members feel unsupported, the burden of responsibility and feelings of isolation may increase (3). Brennan (25) points out the importance of feeling worthy, honoured or esteemed in connection with others, especially in palliative care. This is also in line with our results, where family members expressed dignity as ‘feeling connected to significant others’. Earlier studies have shown that family members have a desire to be seen and have their own needs recognised, but also to be involved in the care (11, 26); however, they cannot be truly involved if they are not included in collaboration at every level (27). This was true in terms of the actual place of care; providing care and being cared for in one’s own home brought a feeling of at‐homeness, as well as a sense of dignity. These results can be linked to those of de Boer et al (28), which showed that family caregivers rated the quality of palliative care higher in the home setting compared to hospice and hospital settings.

Our results revealed that it was important for family members to live as fully as possible. They wanted to do the things they were used to, both as a unique person and with the ill person. However, earlier research has shown this to be a challenge, since they feel that their lives have been put on hold (29). Redefining normal life can be a way for them to come to terms with their new situation (6). Our results show that it is important that healthcare professionals are aware of family members’ needs, in order to support them accordingly. However, in line with Turner et al (26), our study shows that the family members’ own needs are not always recognised by professionals. Being met as an object can seriously hamper both individual and intersubjective dignity. Conversely, being met as a subject by professionals was experienced as being respected as both a unique person and as a dyad (9). Although the word dignity is theoretically used in nursing practice and in governmental documents, the meaning and definition of the term is still unclear, possibly because its understanding is taken for granted. A theoretical concept determination described dignity as embracing an absolute dignity and a relative dignity (30). Absolute dignity included holiness, human worth, freedom, responsibility, duty, and serving one’s fellow men, and being impossible to renounce. Relative dignity is experienced when a person is in a given context and feels harmony with their own ability. This feeling of dignity may be violated, and the person can feel a loss of dignity. Our results show examples of both absolute and relative dignity and suggest possibilities for healthcare professionals to improve the care of family members, supporting their relative dignity and feelings of harmony in the palliative care context. This will also support the dignity of the ill person receiving palliative care.

## Methodological considerations

Readers must judge the trustworthiness of this study based on the transparency of the research process and the cohesion through the whole article (31). The credibility was strengthened by its purposeful sampling (12), including family members who had experiences of the topic and were willing to tell about it, and who had a wide range of ages and relationships to the ill person. However, only three male participants were recruited, which may be considered a limitation. Fifteen participants were considered satisfactory due to the broad variation, with rich in‐depth data of expressions of dignity. One way to support credibility is to identify the most appropriate meaning units; they should be neither too broad nor too narrow (13). We strived to make each meaning unit easy to deal with during condensation and abstraction. Taking all these efforts into consideration with a clear description of the context of the study, transferability to other similar adequate setting are facilitated (31).

## Conclusions and clinical implications

This study contributes to the knowledge of family members’ expressions of dignity. Living with the two‐headed paradox has an impact on their relationship with the ill person and other family members, as well as with healthcare professionals. Living as a respected human being in relation to oneself and to others can be achieved through maintaining one’s identity, feeling connected to others and being comfortable in the situation. Family members’ sense of dignity can be preserved and affected by their own and other persons’s attitudes and behaviours.

Some clinical implications can be suggested how to provide dignified care encompassing the family members. One fundamental caring activity is to invite and encourage them to share their thoughts and feelings of dignity and to acknowledge their competence in the current situation. By being aware of the complexity, and through recognising the reciprocal impact, healthcare professionals can offer tailored support both to family members and to the ill person. In this light, it is of importance for family members not to be trapped in the role of a formal carer. Thus, professionals need to be careful and co‐create caring activities with family members and significant others, enabling them to live as fully as possible. However, further research is needed to explore how the two‐headed paradox impacts all those involved in palliative care, and also on how family members and ill persons affect each other’s sense of dignity, as well as the potential consequences.

## Author contributions

The policy of Scandinavian Journal of Caring Sciences regarding eligibility for authorship has been considered.
